# Correlation of Structure, Tunable Colors, and Lifetimes of (Sr, Ca, Ba)Al_2_O_4_:Eu^2+^, Dy^3+^ Phosphors

**DOI:** 10.3390/ma10101198

**Published:** 2017-10-18

**Authors:** Qidi Xie, Bowen Li, Xin He, Mei Zhang, Yan Chen, Qingguang Zeng

**Affiliations:** 1 School of Applied Physics and Materials, Wuyi University, Jiangmen 529020, China; qidixie@163.com (Q.X.); libowenliboyuan@126.com (B.L.); ychen08@163.com (Y.C.); zengqg1979@126.com (Q.Z.); 2 School of Materials Science and Engineering, Harbin Institute of Technology, Harbin 150001, China

**Keywords:** phosphors, solid solution, average lifetime, AC-LEDs, photoluminescence

## Abstract

(Sr, Ca, Ba)Al_2_O_4_:Eu^2+^, Dy^3+^ phosphors were prepared via a high temperature solid-state reaction method. The correlation of phase structure, optical properties and lifetimes of the phosphors are investigated in this work. For the (Sr, Ca)Al_2_O_4_:Eu^2+^, Dy^3+^ phosphors, the different phase formation from monoclinic SrAl_2_O_4_ phase to hexagonal SrAl_2_O_4_ phase to monoclinic CaAl_2_O_4_ phase was observed when the Ca content increased. The emission color of SrAl_2_O_4_:Eu^2+^, Dy^3+^ phosphors varied from green to blue. For the (Sr, Ba)Al_2_O_4_:Eu^2+^, Dy^3+^ phosphors, different phase formation from the monoclinic SrAl_2_O_4_ phase to the hexagonal BaAl_2_O_4_ phase was observed, along with a shift of emission wavelength from 520 nm to 500 nm. More interestingly, the decay time of SrAl_2_O_4_:Eu^2+^, Dy^3+^ changed due to the different phase formations. Lifetime can be dramatically shortened by the substitution of Sr^2+^ with Ba^2+^ cations, resulting in improving the performance of the alternating current light emitting diode (AC-LED). Finally, intense LEDs are successfully obtained by combining these phosphors with Ga(In)N near UV chips.

## 1. Introduction

AC-LED is a new type of device that can be directly driven by the alternating current of city power. It has experienced rapid development and is widely applied in indoor and outdoor lighting due to its long life, low price, high efficiency, etc. [1,2,3,4].However, the vital defect of the AC-LED is the flickering effect, with a time gap of 5–20 ms [4,5,6], resulting in visual fatigue and health hazards. There have been several solutions to reduce flashing in AC-LED, such as using a converter-free AC-LED driver, increasing drive frequency etc. However, these solutions suffer from low power efficiency or large size. Fortunately, the use of long afterglow phosphors is considered to be a promising method to compensate for the time gap and reduce the flickering effect. Long afterglow phosphors, such as SrSi_2_O_2_N_2_:Eu^2+^, Mn^2+^ [2], SrAl_2_O_4_:Eu^2+^, Ce^3+^, Li^+^ [5], and SrAl_2_O_4_:Eu^2+^, and RE^3+^ (RE = Y, Dy) have been studied and applied to AC-LEDs [4,7].

SrAl_2_O_4_:Eu^2+^ is one of green phosphors for UVLEDs. When it is co-doped with RE^3+^ (RE = Dy, Y, Nd and Ho), SrAl_2_O_4_:Eu^2+^, Re^3+^ can store electrons to achieve long and persistent luminescence. However, the afterglow time of SrAl_2_O4:Eu^2+^, Dy^3+^ is too long to fulfill the requirement of AC-LED. In our previous research, we shortened the average lifetime of SrAl_2_O_4_:Eu^2+^, Dy^3+^ phosphors from 845.86 ms to 428.83 ms by changing the amount of SrCl_2_ flux. Moreover, MAl_2_O_4_:Eu^2+^, Dy^3+^ (M = Ca^2+^, Sr^2+^, Ba^2+^) phosphors with different cations also have different defect levels and afterglow times [7,8,9,10,11,12,13]. Therefore, the substitution of Sr^2+^ by Ca^2+^ or Ba^2+^ ions in SrAl_2_O_4_:Eu^2+^, Dy^3+^ phosphors is expected to tune the afterglow time. Furthermore, the emission and excitation spectra of Eu^2+^ ions can also be modified by the different radius of Ca^2+^, Sr^2+^ and Ba^2+^ ions and crystal structures [13,14,15,16].

This paper aims to improve the emission color and the average lifetime of SrAl_2_O_4_:Eu^2+^, Dy^3+^ phosphors by partially substituting Sr^2+^ with Ca^2+^ or Ba^2+^ ions. The effect and correlation of Ca^2+^ or Ba^2+^ ions substitution on the obtained phases, optical properties, and decay times of SrAl_2_O_4_:Eu^2+^, Dy^3+^ phosphors are systematically investigated. The electroluminescent properties of the as-fabricated LEDs based on UV-chips and MAl_2_O_4_:Eu^2+^, Dy^3+^ (M = Ca^2+^, Sr^2+^, Ba^2+^) phosphors are also investigated.

## 2. Experimental Procedure

Sr_0.90−*x*_Ca*_x_*Al_2_O_4_:0.05Eu^2+^, 0.05Dy^3+^ and Sr_0.90−*y*_Ba*_y_*Al_2_O_4_:0.05Eu^2+^, 0.05Dy^3+^ samples (*x* or *y* = 0.00, 0.15, 0.30, 0.45, 0.60, 0.75, 0.90) were prepared by a high temperature solid-state reaction method. The raw materials CaCO_3_ (99.99%), SrCO_3_ (99.99%), BaCO_3_ (99.99%), Al_2_O_3_ (99.99%), Eu_2_O_3_ (99.99%), Dy_2_O_3_ (99.99%) and H_3_BO_3_ (99.99%) were weighted stoichiometrically. The original materials were mixed into an agate mortar and placed into a small alumina crucible. Then, the mixtures were put into a tubular furnace and heated to 900 °C for 2 h. Being reground, they were heated to 1400 °C for 4 h under a reductive atmosphere from burning activated carbon. Finally, the samples were cooled to room temperature, resulting in Sr_0.9−*x*_Ca*_x_*Al_2_O_4_:0.05Eu^2+^, 0.05Dy^3+^, and Sr_0.9−*y*_Ba*_y_*Al_2_O_4_:0.05Eu^2+^, 0.05Dy^3+^ compounds.

The structure of Sr_0.90−*x*_Ca*_x_*Al_2_O_4_:0.05Eu^2+^, 0.05Dy^3+^, and Sr_0.90−*y*_Ba*_y_*Al_2_O_4_:0.05Eu^2+^, 0.05Dy^3+^ phosphors were explored by X-ray powder diffraction (XRD) using a Diffractometer (X′ Pert PRO, Panalytical, Almelo, Netherlands) with Cu K_α_ radiation at 40 kV and 20 mA. The data were collected in the 2*θ* range of 10° to 80°. The photoluminescence (PL) spectra and decay curves were performed by a spectrofluorometer (F-4600, Hitachi, Tokyo, Japan) equipped with a 150W Xe lamp as the light source.

The phosphors were pre-coated on near-UV Ga(In)N chips with 365 and 395 nm emission, respectively. The emission spectra and parameters of the as-fabricated LEDs were measured by the LED spectrophotocolorimeter (PMS 50, Everfine Co., Ltd., Hangzhou, China) with an integrating sphere of 50 cm diameter. The normal forward-bias current was 20 mA. All measurements were conducted at room temperature.

## 3. Results and Discussion

### 3.1. X-ray Diffraction Analysis

**Figure 1:**
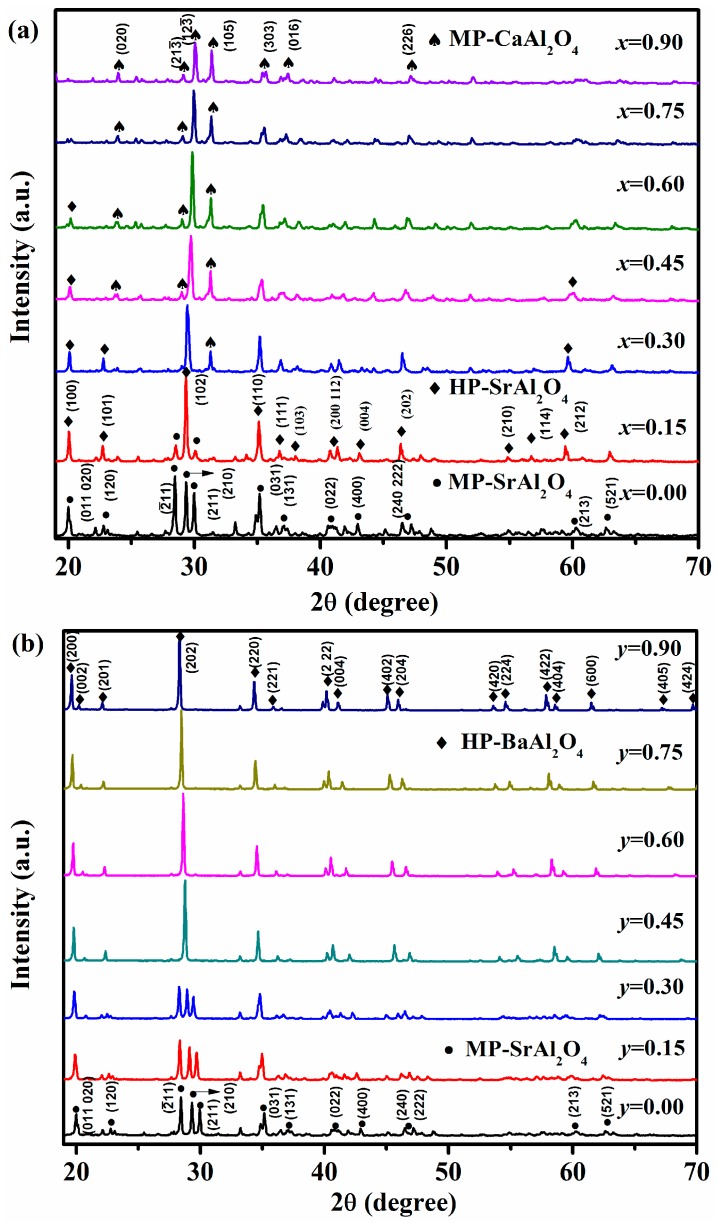
(**a**) X-ray powder diffraction (XRD) patterns of Sr_0.90−*x*_Ca*_x_*Al_2_O_4_:0.05Eu^2+^, 0.05Dy^3+^ phosphors with various *x* (*x* = 0.00, 0.15, 0.30, 0.45, 0.60, 0.75, 0.90); (**b**) XRD patterns of Sr_0.90−*y*_Ba*_y_*Al_2_O_4_:0.05Eu^2+^, 0.05Dy^3+^ phosphors with various *y* (*y* = 0.00, 0.15, 0.30, 0.45, 0.60, 0.75, 0.90).

XRD patterns of Sr_0.90−*x*_Ca*_x_*Al_2_O_4_:0.05Eu^2+^, 0.05Dy^3+^ (*x* = 0.00–0.90) phosphors with various *x* values are depicted in Figure 1a. The patterns are compared with the JCPDS standard cards of possible phases and marked with different symbols. For *x*(Ca^2+^) = 0.00, the diffraction peaks are well matched with the monoclinic SrAl_2_O_4_ (MP-SrAl_2_O_4_, JCPDS card No. 74-0794), and minor content of the raw material—Al_2_O_3_—is observed due to the incomplete reaction, which is explained by our previous work [4]. For *x*(Ca^2+^) = 0.15, the dominant phase is the hexagonal SrAl_2_O_4_ (HP-SrAl_2_O_4_, JCPDS card No. 31-1336). Thus, the different phase formation from the monoclinic to the hexagonal phase happens in Sr_0.90−*x*_Ca*_x_*Al_2_O_4_:Eu^2+^, Dy^3+^ phosphors when the substitution of Sr^2+^ by Ca^2+^ increases from 0.00 to 0.15. The hexagonal phase remains when *x*(Ca^2+^) = 0.30. Simultaneously, the MP-SrAl_2_O_4_ disappears and minor content of monoclinic CaAl_2_O_4_ phase emerges. The monoclinic CaAl_2_O_4_ phase (MP-CaAl_2_O_4_, CPDS card No.53-0191) gradually increases with further elevating concentration of Ca^2+^ from 0.45 to 0.75, accompanying with the decrease of HP-SrAl_2_O_4_. For *x*(Ca^2+^) = 0.90, the dominant phase isMP-CaAl_2_O_4_. In general, as *x*(Ca^2+^) varies from 0.00 to 0.90, and the obtained phases can be varied as follows: MP-SrAl_2_O_4_ → HP-SrAl_2_O_4_ → MP-Cal_2_O_4_. Furthermore, it can be concluded that two different phase always coexist due to the cations substitution except for the samples with *x*(Ca^2+^) = 0.00 and 0.90. The phase change is different with that of other report under different synthesized condition [13].

**Figure 2:**
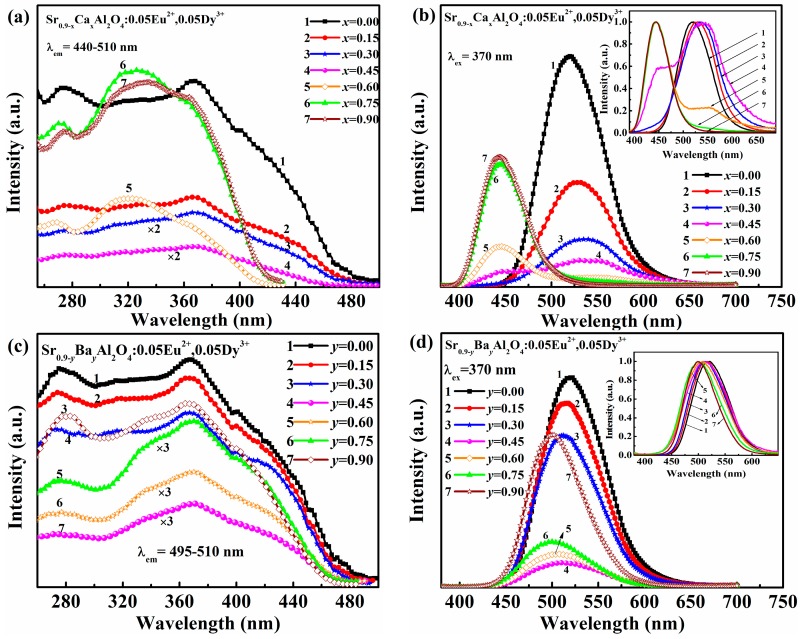
(**a**) Excitation and (**b**) emission spectra of Sr_0.90−*x*_Ca*_x_*Al_2_O_4_:0.05Eu^2+^, 0.05Dy^3+^ phosphors with varied *x* values (Inset is the normalized emission spectra of); (**c**) excitation and (**d**) emission spectra of Sr_0.90−*y*_Ba*_y_*Al_2_O_4_:0.05Eu^2+^, 0.05Dy^3+^ phosphors with varied *y* values (Inset is the normalized emission spectra).

Unlike the substitution of Sr^2+^ by Ca^2+^ in SrAl_2_O_4_:Eu^2+^, Dy^3+^ phosphors, there is only the variation from MP-SrAl_2_O_4_ to hexagonal BaAl_2_O_4_ phase (HP-BaAl_2_O_4_, JCPDS card No. 17-0306) during the substitution of Sr^2+^ by Ba^2+^. The coexistence of two phases cannot be observed from the XRD data, as displayed in Figure 2b. Specifically, when *y*(Ba^2+^) = 0.00–0.30, the crystal structure remains the MP-SrAl_2_O_4_. However, as *y*(Ba^2+^) = 0.45, the XRD patterns are consistent with the standard HP-BaAl_2_O_4_. It remains unchanged while *y* varies from 0.45 to 0.90, which stems from the small radius of Sr^2+^ compared to that of Ba^2+^ and the effect of chemical pressure of Ba^2+^ on the crystal structure. Therefore, the Sr_0.90−*y*_Ba*_y_*Al_2_O_4_:0.05Eu^2+^, 0.05Dy^3+^ compounds can be regarded as the hexagonal BaAl_2_O_4_phase replaced by Sr^2+^ ions as *y*(Ba^2+^) = 0.45–0.90. The different phase formation from MP-SrAl_2_O_4_ to HP-BaAl_2_O_4_ can be obtained when Sr^2+^ is replaced by Ba^2+^ in SrAl_2_O_4_:Eu^2+^, Dy^3+^ phosphors.

Another finding is that the peaks of the Sr_0.90−*x*_Ca*_x_*Al_2_O_4_:0.05Eu^2+^, 0.05Dy^3+^ shift toward larger 2*θ* with Ca^2+^ concentration increased (*x* = 0.45–0.90), while the peaks of the Sr_0.90−*y*_Ba*_y_*Al_2_O_4_:0.05Eu^2+^, 0.05Dy^3+^ shift to smaller 2*θ* when Ba^2+^ concentration increased (*y* = 0.00–0.90). Both of these shifts can be explained by the variation of crystal lattice due to the different ionic radius of Ca^2+^, Sr^2+^, and Ba^2+^ ions of 0.114 nm, 0.123 nm and 0.134 nm, respectively [13,17]. Therefore, when Sr^2+^ is replaced by Ca^2+^, the crystal lattice of SrAl_2_O_4_ phase will shrink. On the contrary, it will expand when Sr^2+^ is substituted by Ba^2+^. Therefore, the difference in the ionic radius leads to the shifts of the diffraction peaks.

### 3.2. Luminescence Properties of Sr_0.09−x_Ca_x_Al_2_O_4_:0.05Eu^2+^, 0.05Dy^3+^ and Sr_0.90−y_Ba_y_Al_2_O_4_:0.05Eu^2+^, 0.05Dy^3+^ Phosphors

Figure 2 illustrates the emission and excitation spectra of Sr_0.90−*x*_Ca*_x_*Al_2_O_4_:0.05Eu^2+^, 0.05Dy^3+^ and Sr_0.90−*y*_Ba*_y_*Al_2_O_4_:0.05 Eu^2+^, 0.05Dy^3+^ phosphors with *x*(*y*) ranged from 0.00 to 0.90. The excitation spectra of Sr_0.90−*x*_Ca*_x_*Al_2_O_4_:0.05Eu^2+^, 0.05Dy^3+^ phosphors are depicted in Figure 2a with monitoring the emission of Eu^2+^ in the range of 440–510 nm. All samples present broad bands from 250 nm to 450 nm due to 4*f*^7^→4*f*^6^5*d*^1^ transitions of Eu^2+^ ions [18]. The shape of excitation bands almost remain unchanged while the intensity decreases when *x* value (*x*= 0.00–0.45) increases. However, it should be noted that excitation bands are different from those of *x* < 0.60. The excitation bands of Sr_0.90−*x*_Ca*_x_*Al_2_O_4_:0.05Eu^2+^, 0.05Dy^3+^ (*x* = 0.60–0.90) are similar and blue-shift, which is ascribed to the phase variation as discussed in XRD analysis. Figure 2b shows the emission spectra of Sr_0.90−*x*_Ca*_x_*Al_2_O_4_:0.05Eu^2+^, 0.05Dy^3+^ phosphors. It can be divided into three sections (*x* = 0–0.30, *x* = 0.45–0.60, *x* = 0.75–0.90). Interestingly, these results are related to the phase transition. First, the luminescence intensity decreases and the emission peak shifts from 520 nm to 538 nm as *x* varied from 0.00 to 0.30. Second, two emission peaks around 450 nm, 540 nm at *x* = 0.45–0.60 are observed due to the coexistence of heterogeneous mixtures of two structures. Finally, the emission spectrum presents a single peak around 444 nm when *x* value increased from 0.75 to 0.90, while the dominant phase is monoclinic CaAl_2_O_4_.

The excitation and emission spectra of Sr_0.90−*y*_Ba*_y_*Al_2_O_4_:0.05Eu^2+^, 0.05Dy^3+^ phosphors are displayed in Figure 2c,d. The excitation spectra show similar outlines with two broad bands around 275 nm, 375 nm and a shoulder around 420 nm. The broad emission bands originate from the typical transitions of 4*f*^7^ → 4*f*^6^5*d*^1^ of Eu^2+^ ions, which are affected by the crystal field of host lattice [19]. The emission band shifts to a shorter wavelength from 520 nm to 500 nm as *y*(Ba^2+^) increased. It can be explained that the crystal field splitting energy is decreased due to the phase change of the substation of Sr^2+^ by the larger Ba^2+^ ions. However, the luminescence intensity decreases when Ba^2+^ content is 0.00–0.45, while it increases when Ba^2+^ content is 0.60–0.90.These changes match very well with the XRD results mentioned above and can be ascribed to Eu^2+^ emission in different phase.

**Figure 3:**
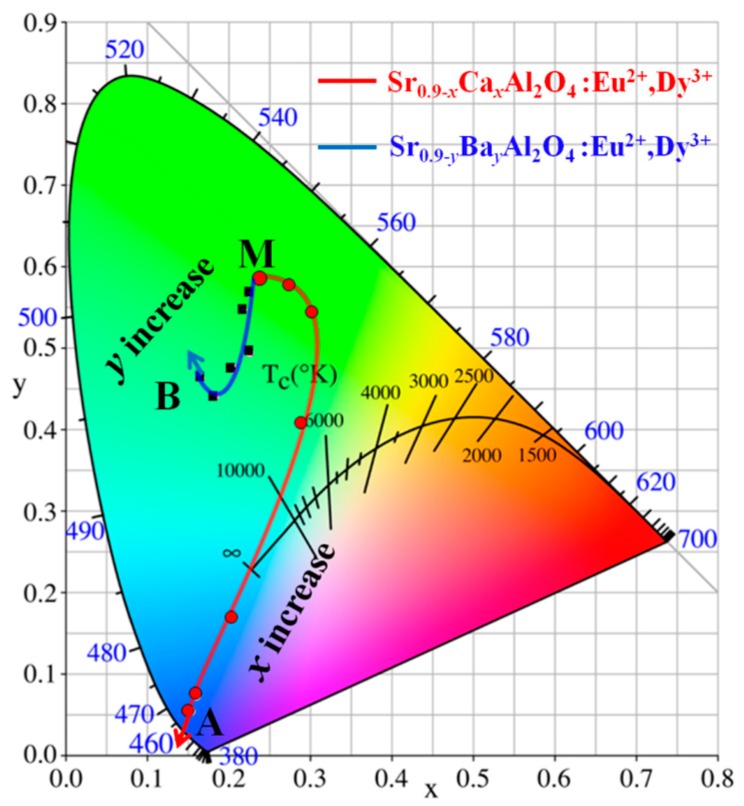
CIE chromaticity diagram of Sr_0.90−*x*_Ca*_x_*Al_2_O_4_:0.05Eu^2+^, 0.05Dy^3+^ and Sr_0.90−*y*_Ba*_y_*Al_2_O_4_:0.05Eu^2+^, 0.05Dy^3+^ phosphors with various *x* (or *y*) values (*x*, *y*= 0.00–0.90).

The CIE chromaticity coordinates calculated from the spectra of Sr_0.90−*x*_Ca*_x_*Al_2_O_4_:0.05Eu^2+^, 0.05Dy^3+^ and Sr_0.9-*y*_Ba*_y_*Al_2_O_4_:0.05Eu^2+^, 0.05Dy^3+^ phosphors with varied *x* (or *y*) values are presented in Figure 3 and Table 1. These results show that the CIE color coordinates of SrAl_2_O_4_:Eu^2+^, Dy^3+^ phosphors can be tuned by Ca^2+^ or Sr^2+^ content. For Sr_0.90−*x*_Ca*_x_*Al_2_O_4_:0.05Eu^2+^, 0.05Dy^3+^ phosphors, the color coordinates can be changed from green (0.2393, 0.5874, point M) to blue (0.1531, 0.0528, point A) area with *x* increased. In addition, the color coordinates of Sr_0.9−*y*_Ba*_y_*Al_2_O_4_:0.05Eu^2+^, 0.05Dy^3+^ phosphors have a little shift from green (0.2399, 0.5867, point M) to bluish green (0.1652, 0.4645, point B) area. The above discussion indicates that the emission color of SrAl_2_O_4_:Eu^2+^, Dy^3+^ can be tuned due to the phase change of the substitution of Sr^2+^ with Ca^2+^ or Ba^2+^ ions. It is an important challenge to tune the color of phosphors for improving the color rendering index of w-LEDs [20,21,22].

### 3.3. Decay Characteristics

**Figure 4:**
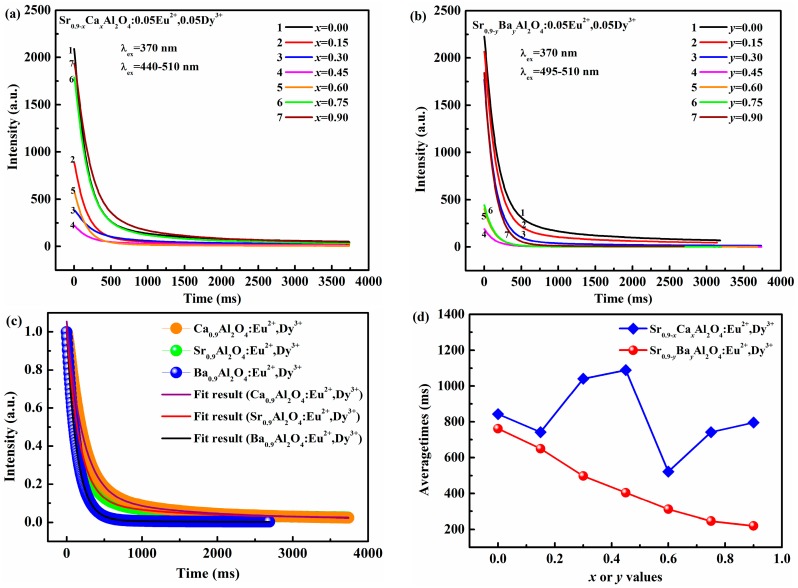
(**a**) Photoluminescence decay curves of Sr_0.90−*x*_Ca*_x_*Al_2_O_4_:0.05Eu^2+^, 0.05Dy^3+^ with various *x*(Ca^2+^) (λ_ex_ = 370 nm λ_em_ = 440–510 nm); (**b**) photoluminescence decay curves of Sr_0.90−*y*_Ba*_y_*Al_2_O_4_:0.05Eu^2+^, 0.05Dy^3+^ with various *y*(Ba^2+^) (λ_ex_ = 370 nm λ_em_ = 495–510 nm); (**c**) normalized decay and fitted curves of phosphors (Ca_0.90_Al_2_O_4_:0.05Eu^2+^, 0.05Dy^3+^, Sr_0.90_Al_2_O_4_:0.05Eu^2+^, 0.05Dy^3+^, Ba_0.90_Al_2_O_4_:0.05Eu^2+^, 0.05Dy^3+^); (**d**) average lifetimes of Sr_0.90−*x*_Ca*_x_*Al_2_O_4_:0.05Eu^2+^, 0.05Dy^3+^, and Sr_0.90−*y*_Ba*_y_*Al_2_O_4_:0.05Eu^2+^, 0.05Dy^3+^.

The photoluminescence decay curves of SrAl_2_O_4_:Eu^2+^, Dy^3+^ phosphors with varied Ca^2+^ and Ba^2+^ content are shown in Figure 4a,b, respectively. The figures reveal the initial luminous intensity is different when Sr^2+^ is replaced by either Ca^2+^ or Ba^2+^ in SrAl_2_O_4_:Eu^2+^, Dy^3+^.Among these phosphors, Sr_0.90_Al_2_O_4_:0.05Eu^2+^, 0.05Dy^3+^ has the highest initial luminous intensity. The initial luminous intensity gradually decreases when the content of Ca^2+^(Ba^2+^) increased to 0.45. By further increasing the Ca^2+^ or Ba^2+^ concentration, the initial luminous intensity enhances. The variation of initial luminous intensity is consistent with the emission spectra, which can be explained by the change of crystal structure.

Moreover, the decay process of all samples is composed of fast decay, medium decay and subsequent of slow decay, which is consistent with our previous report. The decay curves of Ca_0.90_Al_2_O_4_:0.05Eu^2+^, 0.05Dy^3+^, Sr_0.90_Al_2_O_4_:0.05Eu^2+^, 0.05Dy^3+^, and Ba_0.90_Al_2_O_4_:0.05Eu^2+^, 0.05Dy^3+^ phosphors are fitted as representatives, which are presented in Figure 4c. The decay curves are well fitted with the following triple exponential functions:

(1) $$I(t)=I_{{}_{0}}+Ae^{-t/\tau_{1}}+Be^{-t/\tau_{2}}+Ce^{-t/\tau_{3}}$$

where *t* is the time, *I_0_* and *I* is the luminescence intensity at initial time and *t*, respectively. *A*, *B* and *C* are constants. τ_1_, τ_2_, and τ_3_ are the decay time for the exponential components, respectively. The average lifetimes can be obtained as follows. The detail parameters are listed in Table 2.

(2) $$\tau_{average}=\frac{\int_{0}^{\infty }t\times I_{\left(\tau \right)}dt}{\int_{0}^{\infty }I_{\left(t\right)}dt}$$

On the basis of the Equation (2), the average lifetime (τ_average_) of SrAl_2_O_4_:Eu^2+^, Dy^3+^ phosphors with varied Ca^2+^ and Ba^2+^ contents are determined and shown in Figure 4d, respectively. The order of the average lifetimes is Sr_0.90_Al_2_O_4_:0.05Eu^2+^, 0.05Dy^3+^ > Ca_0.90_Al_2_O_4_:0.05Eu^2+^, 0.05Dy^3+^ > Ba_0.90_Al_2_O_4_:0.05Eu^2+^, 0.05Dy^3+^. The average lifetimes of Sr_0.90−*x*_Ca*_x_*Al_2_O_4_:0.05Eu^2+^, 0.05Dy^3+^ phosphors vary with the change of Ca^2+^ content, and the maximum is about 1088.5 ms at *x* = 0.45. It could be ascribed to the complicate formation of the phase in Figure 1. Furthermore, the average lifetimes of Sr_0.90−*y*_Ba*_y_*Al_2_O_4_:0.05Eu^2+^, 0.05Dy^3+^ phosphors gradually decrease with Ba^2+^ content increased due to the different phase formation and reach the shortest time of 219.91 ms at *y* = 0.90, demonstrating that the average lifetime or decay characteristics of SrAl_2_O_4_:Eu^2+^. Dy^3+^ could be adjusted by the partial substitution of Ba^2+^, Ca^2+^. The results also indicate that the tuned lifetimes are more suitable for the application in AC-LEDs as the global widely utilize 50/60 Hz alternating current with a 5–20 ms dark duration.

### 3.4. Application of SrAl_2_O_4_:Eu^2+^, Dy^3+^ Phosphors with Varied Contents of Ca^2+^ and Ba^2+^ in LEDs

Finally, phosphors converted LEDs were fabricated with silicone, near UV-chips (~365 nm, 395 nm), Sr_0.90−*x*_Ca*_x_*Al_2_O_4_:0.05Eu^2+^, 0.05Dy^3+^, and Sr_0.90−*y*_Ba*_y_*Al_2_O_4_:0.05Eu^2+^, 0.05Dy^3+^ phosphors. The phosphors account for 20% of the mass of silicone.

**Figure 5:**
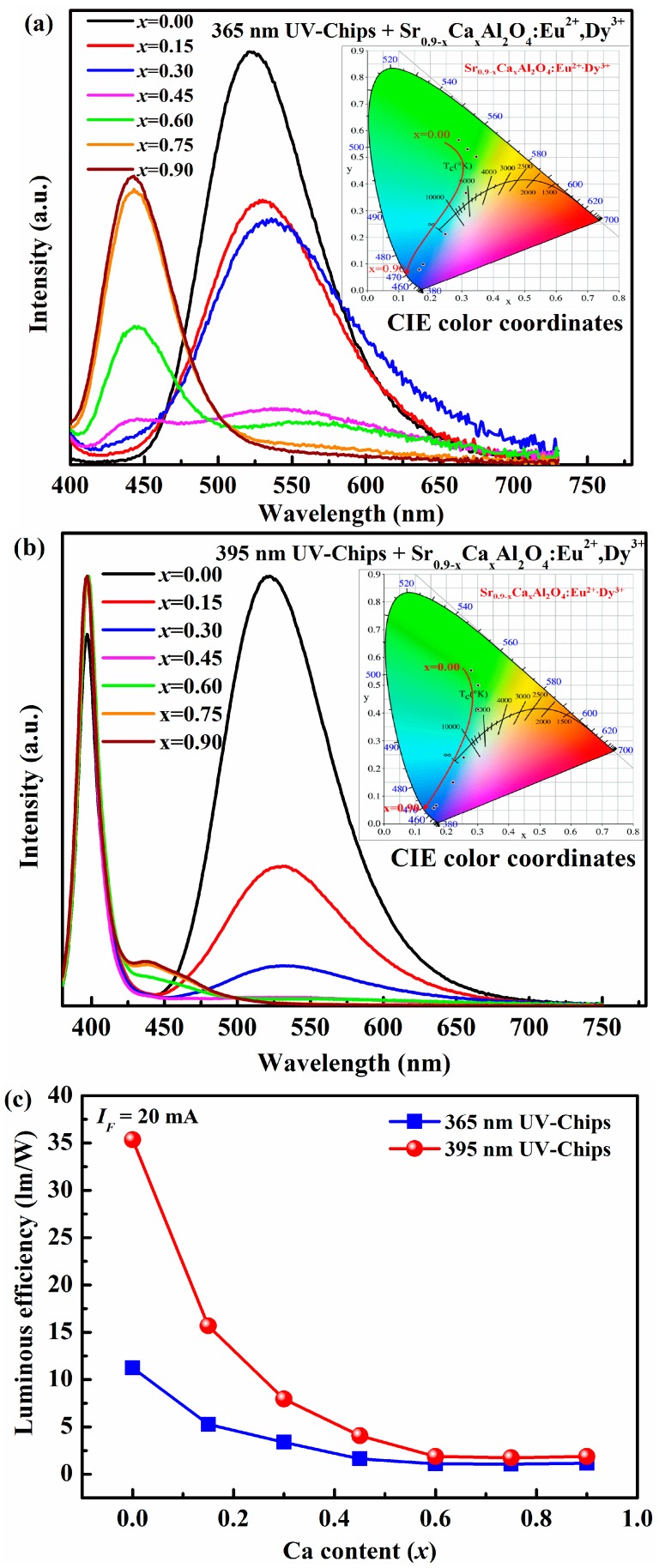
Electroluminescence spectra of the as-fabricated LEDs based on UV chip and Sr_0.90−*x*_Ca*_x_*Al_2_O_4_:0.05Eu^2+^, 0.05Dy^3+^ phosphors under 20 mA forward-bias current (i.e., *I_F_* = 20 mA). Inset is chromaticity diagram of the as-fabricated LEDs: (**a**) 365 nm UV chip; (**b**) 395 nm UV-chip; (**c**) Luminous efficiency of as-fabricated LEDs with different Ca^2+^ content under *I_F_* = 20 mA.

Figure 5 is the electroluminescence spectra of the as-fabricated LEDs based on Sr_0.90−*x*_Ca*_x_*Al_2_O_4_:0.05Eu^2+^, 0.05Dy^3+^ phosphors and near UV Ga(In)N chips. In the Figure 4a, the shapes of all electroluminescence spectra in the as-fabricated LEDs are similar with the photoluminescence emission spectra of Sr_0.90−*x*_Ca*_x_*Al_2_O_4_:0.05Eu^2+^, 0.05Dy^3+^ phosphors because the near UV around 365 nm of chips is completely absorbed by phosphors. Figure 5b depicts that the emission band located at 395 nm belongs to the UV-chips. And other emission bands are ascribed to Eu^2+^ emissions excited by 395 nm UV-chips. The results prove that the Sr_0.90−*x*_Ca*_x_*Al_2_O_4_:0.05Eu^2+^, 0.05Dy^3+^ fluorescence materials can absorb the light with wavelength of 365 nm or 395 nm, and convert it into green or blue visible-light. The CIE color coordinates of as-fabricated LEDs are calculated and listed in Table 3. The inset of Figure 5 presents the chromaticity diagram of the as-fabricated LEDs based on SrAl_2_O_4_:Eu^2+^, Dy^3+^ phosphors, which can be tuned from green to blue area and are similar with that of phosphors. In addition, the luminous efficiency is also systematically investigated under 20 mA forward-bias current in this work, as indicated in Figure 5c. The luminous efficiency of the as-fabricated LEDs with 395 nm is higher than in the case of 365 nm UV chips. However, for both kinds of LEDs fabricated by 365 nm and 395 nm UV-chips, the luminous efficiency decreases strongly, then increases slowly with Ca^2+^ content elevated. The maximum luminous efficiency is 11.24 lm/W for 365 nm LEDs and 35.35 lm/W for 395 nm LEDs (*x* = 0).

**Figure 6:**
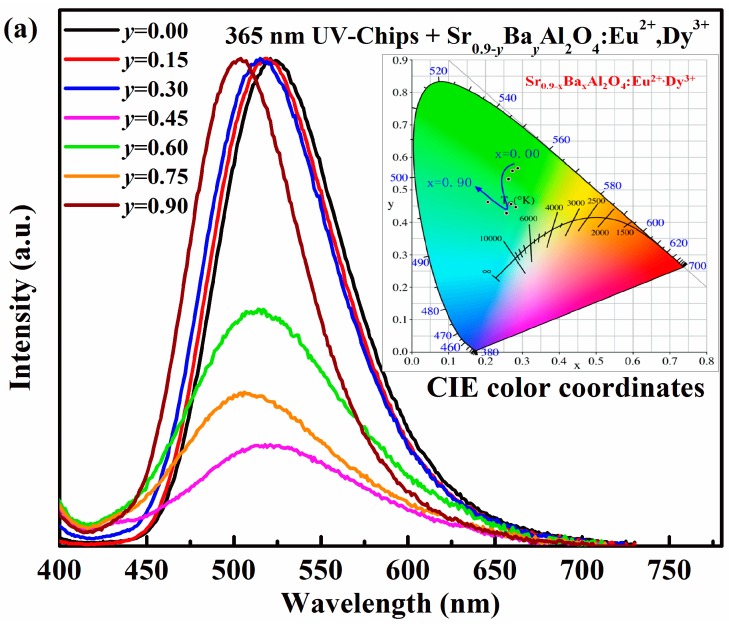

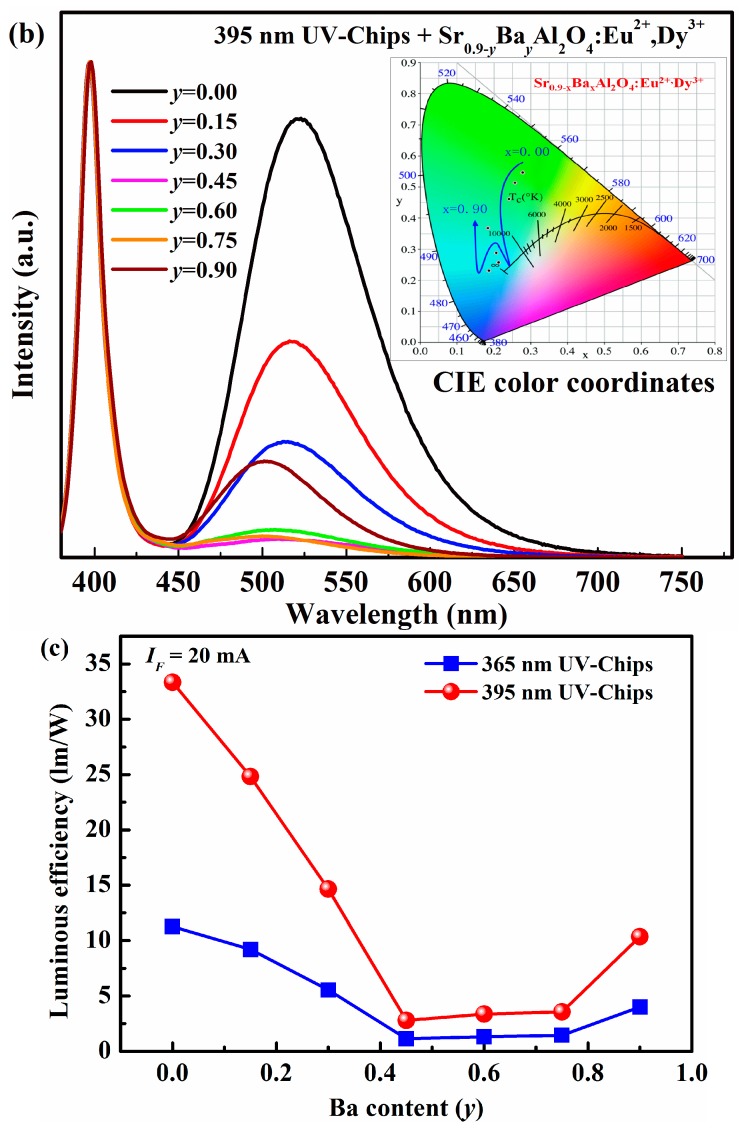
Electroluminescence spectra of the as-fabricated LEDs based on UV-chip and Sr_0.90−*y*_Ba*_y_*Al_2_O_4_:0.05Eu^2+^, 0.05Dy^3+^ phosphors under 20 mA forward-bias current (i.e., *I_F_* = 20 mA). Inset is chromaticity diagram of the as-fabricated LEDs: (**a**) 365 nm UV-chip; (**b**) 395 nm UV-chip; (**c**) Luminous efficiency of as-fabricated LEDs with different Ba^2+^ content under *I_F_* = 20 mA.

The electroluminescence spectra of LEDs based on Sr_0.90−*x*_Ba*_x_*Al_2_O_4_:0.05Eu^2+^, 0.05Dy^3+^ phosphors are also investigated, as shown in Figure 6.Except emission peaks of the UV chip itself, all of the electroluminescence spectra of Sr_0.90−*y*_Ba*_y_*Al_2_O_4_:0.05Eu^2+^, 0.05Dy^3+^ excited by both 365 nm and 395 nm UV-chips are similar. It indicates that the Sr_0.9-*y*_Ba*_y_*Al_2_O_4_:Eu^2+^, Dy^3+^ phosphors can absorb 365 nm or 395 nm emission from Ga(N)In chips, and convert it into green or blue-green visible-light. The CIE color coordinates of the as-fabricated LEDs are calculated and listed in Table 4. By varying Ba^2+^ concentrations, the color of the as-fabricated LEDs can be tuned, but the adjustable zone is narrower than in the case of Ca^2+^. It suggests that the color coordinates of pc-LEDs cannot be influenced by near UV Ga(In)N chips. However, the luminous efficiency decreases at first, and then increases with Ba^2+^ content enhanced. The maximum luminous efficiency is 11.26 lm/W for 365 nm LEDs and 33.34 lm/W for 395 nm LEDs (*y* = 0). The variation of luminous efficiency of the as-fabricated LED is also consistent with the emission spectra due to the different phase formation.

## 4. Conclusions

Sr_0.90−*x*_Ca*_x_*Al_2_O_4_:0.05Eu^2+^, 0.05Dy^3+^ and Sr_0.90−*y*_Ba*_y_*Al_2_O_4_:0.05Eu^2+^, 0.05Dy^3+^ (*x* or *y* = 0.00, 0.15, 0.30, 0.45, 0.60, 0.75, 0.90) phosphors were synthesized. The variation of optical properties for the phosphors is ascribed to the phase structures. For the series of Sr_0.90−*x*_Ca*_x_*Al_2_O_4_:0.05Eu^2+^, 0.05Dy^3+^ phosphors, two heterogeneous structures coexist, except the samples with *x*(Ca^2+^) = 0.00 and 0.90. The emission color of SrAl_2_O_4_:Eu^2+^, Dy^3+^ phosphor can be adjusted largely from green to blue, and the lifetime can also be tuned largely from 1088.5 ms to 521.6 ms. For Sr_0.90−*y*_Ba*_y_*Al_2_O_4_:0.05Eu^2+^, 0.05Dy^3+^ phosphors, the phase transforms from monoclinic SrAl_2_O_4_ to hexagonal BaAl_2_O_4_ phase. Meanwhile, the emission wavelength shifts from 520 nm to 500 nm, and the color slightly changes from green to blue-green. More importantly, the average lifetime of SrAl_2_O_4_:Eu^2+^, Dy^3+^ can be shortened from 842.04 ms to 219.91 ms, which can appropriately compensate for the AC time gap. Finally, the LEDs are successfully fabricated by combining the phosphors with Ga(In)N UV-chips. The maximum of luminous efficiency reaches 33.34 lm/W based on SrAl_2_O_4_:Eu^2+^, Dy^3+^ phosphors.

## Figures and Tables

**Table 1:** Color coordinates of Sr_0.90−*x*_Ca*_x_*Al_2_O_4_:0.05Eu^2+^, 0.05Dy^3+^ and Sr_0.90−*y*_Ba*_y_*Al_2_O_4_:0.05Eu^2+^, 0.05Dy^3+^ phosphors (*x*, *y* = 0.00–0.90).

*x* (or *y*) Values	Sr_0.90−*x*_Ca*_x_*Al_2_O_4_:0.05Eu^2+^, 0.05Dy^3+^		Sr_0.90−*y*_Ba*_y_*Al_2_O_4_:0.05Eu^2+^, 0.05Dy^3+^	
	CIE (*x*, *y*)		CIE (*x*, *y*)	
	*x*	*y*	*x*	*y*
0.00	0.2393	0.5874	0.2399	0.5867
0.15	0.2741	0.5754	0.2251	0.5675
0.30	0.3029	0.5456	0.2170	0.5477
0.45	0.2860	0.4086	0.2258	0.4952
0.60	0.2059	0.1712	0.2054	0.4741
0.75	0.1607	0.0729	0.1823	0.4412
0.90	0.1531	0.0528	0.1652	0.4645

**Table 2:** Results for fitted decay curve of Ca_0.90__−*x*_Al_2_O_4_:0.05Eu^2+^, 0.05Dy^3+^, Sr_0.90__−*x*_Al_2_O_4_:0.05Eu^2+^, 0.05Dy^3+^, and Ba_0.90__−*x*_ Al_2_O_4_:0.05Eu^2+^, 0.05Dy^3+^ phosphors.

Samples	Decay Lifetimes (ms)			
	τ_1_	τ_2_	τ_3_	τ_average_
Ca_0.90_Al_2_O_4_:0.05Eu^2+^, 0.05Dy^3+^	210.28	210.28	1410.88	795.11
Sr_0.90_Al_2_O_4_:0.05Eu^2+^, 0.05Dy^3+^	1264.65	165.05	165.05	842.04
Ba_0.90_Al_2_O_4_:0.05Eu^2+^, 0.05Dy^3+^	134.50	1502.09	134.50	219.91

**Table 3:** Parameter of chromaticity coordinates based on Sr_0.90−*x*_Ca*_x_*Al_2_O_4_:0.05Eu^2+^, 0.05Dy^3+^ phosphors with 365 nm and 395 nm UV-Chips under 20 mA forward-bias current.

*x* Values	395 UV-Chips		365 UV-Chips	
	CIE (*x*, *y*)		CIE (*x*, *y*)	
	*x*	*y*	*x*	*y*
0.00	0.2805	0.5527	0.2907	0.5657
0.15	0.3029	0.5005	0.3181	0.5301
0.30	0.3033	0.4120	0.3462	0.5032
0.45	0.2555	0.2402	0.3139	0.3662
0.60	0.2209	0.1497	0.2486	0.2125
0.75	0.1675	0.0669	0.1771	0.0983
0.90	0.1599	0.0572	0.1655	0.0791

**Table 4:** Parameter of chromaticity coordinates based on Sr_0.90−*x*_Ba*_x_*Al_2_O_4_:0.05Eu^2+^, 0.05Dy^3+^ phosphors with 365 nm and 395 nm UV-Chips under 20 mA forward-bias current.

*y* Values	395 UV-Chips		365 UV-Chips	
	CIE (*x*, *y*)		CIE (*x*, *y*)	
	*x*	*y*	*x*	*y*
0.00	0.2782	0.5471	0.2879	0.5666
0.15	0.2569	0.5045	0.2735	0.5584
0.30	0.2412	0.4612	0.2627	0.5331
0.45	0.2127	0.2568	0.2825	0.4471
0.60	0.2071	0.2881	0.2693	0.4560
0.75	0.1864	0.2309	0.2580	0.4278
0.90	0.1839	0.3678	0.2070	0.4628
